# Intertwining of Activin A and TGFβ Signaling: Dual Roles in Cancer Progression and Cancer Cell Invasion

**DOI:** 10.3390/cancers7010070

**Published:** 2014-12-30

**Authors:** Holli A. Loomans, Claudia D. Andl

**Affiliations:** 1 Department of Cancer Biology, Vanderbilt University Medical Center, Nashville, TN 37232, USA; E-Mail: holli.a.loomans@vanderbilt.edu; 2 Department of Surgery, Vanderbilt University Medical Center, Nashville, TN 37232, USA; 3 Vanderbilt Ingram Cancer Center, Vanderbilt University Medical Center, Nashville, TN 37232, USA; 4 Vanderbilt Digestive Disease Center, Vanderbilt University Medical Center, Nashville, TN 37232, USA; 5 Vanderbilt Epithelial Biology Center, Vanderbilt University Medical Center, Nashville, TN 37232, USA

**Keywords:** TGFβ, activin A, cell adhesion, cell migration, EMT, cancer

## Abstract

In recent years, a significant amount of research has examined the controversial role of activin A in cancer. Activin A, a member of the transforming growth factor β (TGFβ) superfamily, is best characterized for its function during embryogenesis in mesoderm cell fate differentiation and reproduction. During embryogenesis, TGFβ superfamily ligands, TGFβ, bone morphogenic proteins (BMPs) and activins, act as potent morphogens. Similar to TGFβs and BMPs, activin A is a protein that is highly systemically expressed during early embryogenesis; however, post-natal expression is overall reduced and remains under strict spatiotemporal regulation. Of importance, normal post-natal expression of activin A has been implicated in the migration and invasive properties of various immune cell types, as well as endometrial cells. Aberrant activin A signaling during development results in significant morphological defects and premature mortality. Interestingly, activin A has been found to have both oncogenic and tumor suppressor roles in cancer. Investigations into the role of activin A in prostate and breast cancer has demonstrated tumor suppressive effects, while in lung and head and neck squamous cell carcinoma, it has been consistently shown that activin A expression is correlated with increased proliferation, invasion and poor patient prognosis. Activin A signaling is highly context-dependent, which is demonstrated in studies of epithelial cell tumors and the microenvironment. This review discusses normal activin A signaling in comparison to TGFβ and highlights how its dysregulation contributes to cancer progression and cell invasion.

## 1. Introduction

As a growing body of research has unraveled the functional consequences of transforming growth factor β (TGFβ) superfamily signaling, it has also revealed the complexity of these signaling networks and their pleiotropic effects. This family of ligands consists of TGFβ, bone morphogenic proteins (BMPs), activins and anti-Müllerian hormone (AMH) proteins. Of these proteins, TGFβ and BMPs have been well characterized for their roles in development, angiogenesis and epithelial-to-mesenchymal transition (EMT) during cancer, particularly regarding cell migration and invasion [1,2,3,4,5,6,7]. Conversely, activin A signaling is less well understood. Activins are homo- or hetero-dimers of activin β subunits. Currently, there are three known bioactive activin dimers: activins A (β_A_β_A_), B (β_B_β_B_) and AB (β_A_β_B_) [8,9,10,11,12,13,14,15]. Activin A is best understood for its function in embryogenesis and reproduction; however, its role during cancer progression is still not well documented. This review focuses on the current understanding of normal activin A signaling, the functional similarities and differences between activin A and TGFβ and how this signaling pathway becomes dysregulated during cancer progression, influencing cell migration and invasion. Understanding the regulatory mechanism of activin A in cell migration and invasion will allow for better insight into its role in cancer initiation and progression.

## 2. Signaling Regulation

### 2.1. Activin A and TGFβ Signaling

Though activin A and TGFβ show structural similarity, activin A is secreted as an active protein, whereas TGFβ is secreted as an inactive precursor and requires activation [16]. Multiple proteins have been identified that can activate latent TGFβ, including proteolytic processing by plasmin and cathepsin D, as well as nonproteolytic processing by heat and detergents [17].

Not only do TGFβ superfamily ligands share structural similarity (they share six to nine cysteine residues that form disulfide bonds), the receptor complexes often overlap ligand specificity and downstream signal transduction [18]. TGFβ receptor complexes are heteromeric complexes that consist of a type I and type II receptor homodimer [19]. Type II and I receptors are distinguished by their sequence. Type II receptors are constitutively active transmembrane serine/threonine kinases [20]. These receptors initially bind a TGFβ superfamily ligand to recruit a type I receptor and begin the signal transduction cascade. The number of known type II receptors is limited: transforming growth factor β receptor II (TGFβRII) preferentially binds TGFβ; bone morphogenic protein receptor II (BMPRII) is known to bind multiple ligands, including inhibin A; activin receptor type II and IIB (ActRII/IIB) bind several ligands, of particular interest activin A, inhibin A/B, and nodal; and the Müllerian inhibitory substance type II receptor (MISRII), which is only known to bind AMH (summarized in [21]). Type I receptors, commonly termed activin receptor-like kinases (ALKs), contain highly-conserved kinase domains and a glycine-serine rich juxtamembrane domain, a necessary component for their phosphorylation and activation [22,23]. To date, seven ALKs (ALK1-7) are known and have been characterized. These receptors have been succinctly summarized elsewhere [21]. TGFβ has been shown to preferentially signal through ALK5 (TβRI), while activin A signals primarily via ALK4 [24]. Interestingly, ALK5 and ALK4 show almost identical kinase domains; however, they dimerize with different type II receptors [25].

**Figure 1:**
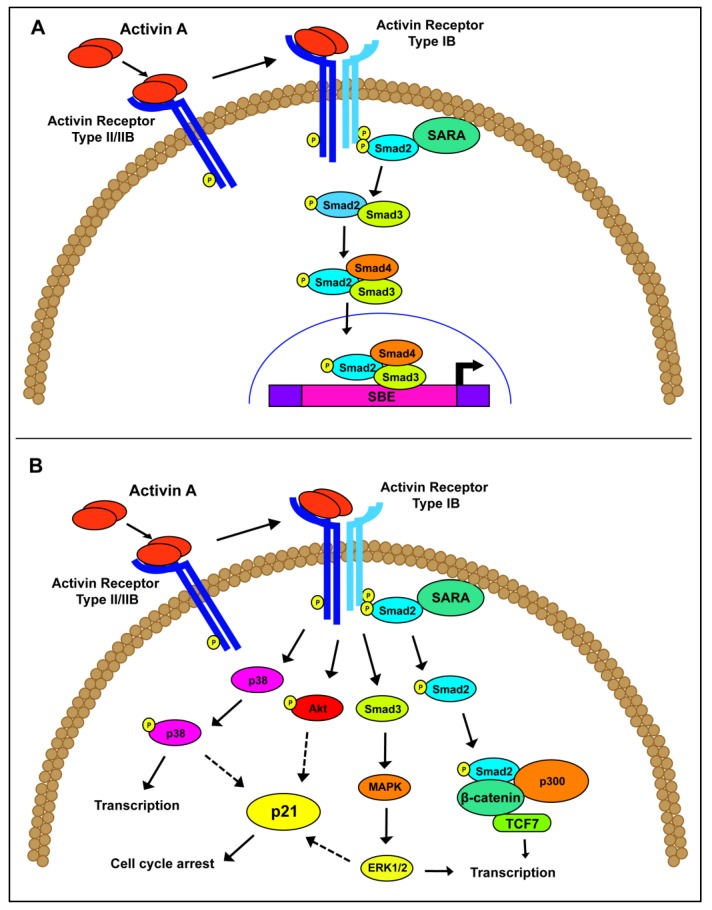
Schematic of activin A signaling. (**A**) Canonical activin A signaling occurs through the phosphorylation and activation of the Smad proteins, leading to downstream gene transcription. (**B**) Non-canonical activin A signaling has been postulated through a variety of pathways, including P13K/Akt, MAPK/ERK and β-catenin/p300.

Focusing specifically on activin A signaling, the signaling pathway begins with an active activin A ligand secreted from the cell. Activin A binds to ActRII/IIB, which recruits a type I receptor, preferentially ALK4, to form a signal transducing heterodimer [9,26,27,28]. In a similar mechanism to TGFβ signaling, Smad2/3 is recruited and phosphorylated by ALK4. Active Smad2/3 is released into the cytoplasm and complexes with the co-Smad, Smad4. This complex translocates to the nucleus, where it binds to Smad binding elements, with the consensus sequence CAGA, to drive transcription of downstream effectors (Figure 1A). In addition to this canonical activin A pathway, non-canonical signaling, such as Akt/PI3K, MAPK/ERK and Wnt/β-catenin, have been associated with activin A function independently of Smad activation (Figure 1B) [29,30]. Despite their overlapping Smad-dependent or non-canonical pathways, activin A and TGFβ operate through common, as well as distinct, downstream transcriptional targets, resulting in different functional consequences [31,32,33,34,35].

Due to the overlapping signal transduction pathway of activin A and TGFβ, it is difficult to untangle specific downstream transcriptional targets for the respective pathways. The best-defined differential downstream targets of activin A and TGFβ signaling are in the context of human embryonic stem cells (hESCs). In hESCs, activin A drives downstream transcription of Nanog, whereas TGFβ signaling in this context induces the transcription of SRY-box 2 (Sox2) and octamer binding protein-4 (Oct4) to induce self-renewal and differentiation, which is negatively regulated through the activation of BMP signaling [36,37,38,39]. In adult tissues, discerning between activin A and TGFβ signaling is more difficult. Both activin A and TGFβ, via the Smad2/3/4 complex, have been shown to regulate various cell cycle and extracellular matrix proteins, such as p15, plasminogen activator-1 (PAI-1) and collagen I [40]. With the advent of new methodologies, future research should focus on unweaving differential activin A and TGFβ signaling in post-natal tissues.

### 2.2. Mechanism of Activin A Regulation

**Figure 2:**
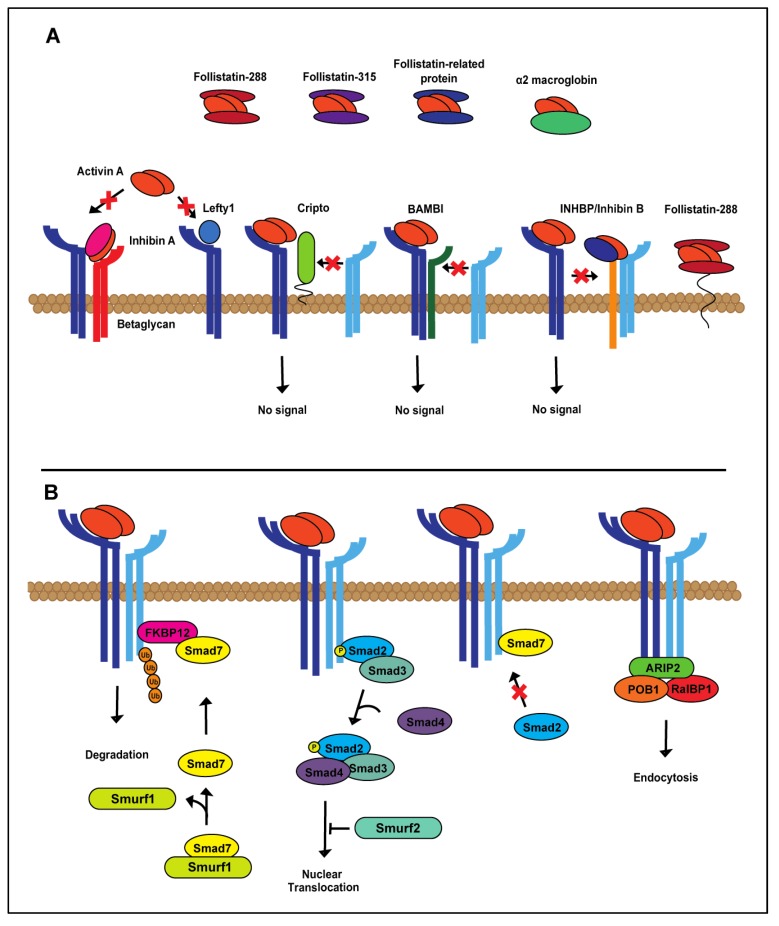
Extra- and intra-cellular regulation of activin A signaling. (**A**) Activin A is tightly regulated at the extracellular level by both extracellular matrix (follistatin-288, follistatin-315, follistatin-related protein, α2-macroglobin, left-right determination factor 1 (Lefty1), inhibin A) and membrane-bound proteins (betaglycan, Cripto, BAMBI, INHBP/inhibin B, follistatin-288). (**B**) In the cytoplasm, canonical activin A signaling is controlled during each step of the Smad cascade.

Activin A expression is tightly regulated. Regulators of the activin A signaling cascade can be found in the extracellular matrix, at the cell membrane and intracellularly (Figure 2A,B) [41,42,43]. As there are numerous mechanisms of activin A regulation, we have focused specifically on the most studied, best understood mechanisms of activin A regulation, follistatin and inhibin A, endogenous inhibitors found at the cell membrane and in circulation [44,45,46,47]. Follistatin is expressed in three forms, follistatin-288, follistatin-303 and follistatin-315 [48,49], with different modes of action (Figure 2A). Two follistatin proteins surround and inhibit activin A by blocking access to both the activin receptor type I and II binding sites [45,50,51]. Follistatin-288, which is mainly found at the cell membrane, sequesters activin A, resulting in endocytosis and lysosomal degradation [1,2,3,4,5,6,7,51,52,53,54]. Follistatin-288 function itself can be regulated through cleavage at the cell surface by heparin and function in circulation [8,9,10,11,12,13,14,15,51,54,55]. Follistatin-303 is produced in relatively low abundance compared to the other follistatin isoforms and has moderate affinity for cell surface proteoglycans, but can bind activin A in circulation, as well as at the cell membrane [1,9,26,27,28,37,49]. The effects of activin A signaling can also be counteracted by a structurally-related ligand, inhibin A. Inhibin A is a heterodimer composed of inhibin α and β_A_ subunits [29,30,56,57]. Inhibin A, bound to the transmembrane receptor, betaglycan (TBRIII), dimerizes with ActRII/IIB, preventing activin A binding [31,32,33,34,35,58,59]. TGFβ signaling demonstrates similar levels of regulation, indicating the importance of maintaining the signaling balance of this superfamily [2,4,6,41,42,43,60,61]. Additional mechanisms of activin A regulation on the extra- and intra-cellular levels are shown in Figure 2.

## 3. Hijacked Developmental Processes and Their Contributions in Tumorigenesis

### 3.1. Early Development and Stem Cell Biology

Embryonic development requires particular populations of cells to undergo EMT, migration and complete implantation and gastrulation [44,45,46,47,56,62]. Activin A plays a significant part in this process. Initially described as XTC-MIF, activin A was found to be a potent morphogen and inducer of the mesodermal patterning [11,13,15,26,48,49,63,64]. With increasing concentrations, activin A can induce mesodermal cell differentiation, inducing epidermal cell fate at the lowest concentrations, as well as Spemann organizer patterning at the highest concentrations [9,26,27,28,45,50,51,61,65,66]. Spatial patterning occurs via a diffusible activin A concentration gradient in the extracellular matrix (ECM) [29,67]. Interestingly, TGFβ plays a minor role in these developmental processes. Similar to activin A, TGFβ diffuses along a similar gradient; however, it does not induce the same spectrum of cell fates as activin A does and requires a much higher concentration of TGFβ ligand [31,51,54,68].

In human development, activin A is necessary to maintain pluripotency and the subsequent differentiation of human pluripotent stem cells (hPSC) [50,69]. Prolonged treatment of hPSC with activin A induces definitive endoderm differentiation [52,70]. During early hPSC differentiation, activin/nodal signaling is critical to induce epithelial-mesodermal switching, indicated by the loss of the epithelial marker, CD326 (epithelial cell adhesion molecule, EPCAM), and upregulation of mesodermal marker CD56 (neural cell adhesion molecule 1, NCAM1) [55,59,71]. Activin A, in concert with Nodal, signals through Smad2/3 to induce Nanog expression, which is necessary to maintain the expression of genes involved in pluripotency [1,3,7,37]. Maintaining pluripotency in cells is necessary, not only to achieve proper development, but also to initiate and sustain tumorigenesis. Activin A has been shown to be necessary for the maintenance of self-renewal in human embryonic stem cells through the induction of Oct4, Nanog, nodal and wingless-type MMTV integration site family member 3 (Wnt3) [36] and, more importantly, the induction of basic fibroblast growth factor 2 (FGF-2) and suppression of BMP7 [72]. Suppression of the downstream target inhibitor of DNA binding 2 (Id2) by activin A and TGFβ is central in the induction of EMT, which is antagonized by BMPs [73]. Of interest, EMT is associated with the acquisition of malignant traits and stem cell markers, therefore linking TGFβ signaling to the regulation of cancer stem cells [74].

### 3.2. EMT vs. Collective Migration

Different members of the TGFβ superfamily (TGFβ1, TGFβ2, TGFβ3, activin A or BMP7) have been analyzed for their potential to induce EMT in epithelial cells of different origins. While TGFβ1, TGFβ2 and TGFβ3 induce characteristic features of EMT in the mammary and lung cells along with the downregulation and delocalization of E-cadherin, activin A does not induce EMT in mammary cells or keratinocytes, but causes the scattering and spindle-like morphology of lung cells [75]. As EMT is widely recognized to lead to increased cell invasion, loss of E-cadherin is a hallmark of EMT [76]. TGFβ participates in the EMT process through the regulation of transcription factors, such as Snail family zinc finger 1 (Snail), zinc finger E-box binding homeobox 1(ZEB) and twist family BHLH transcription factor 1 (twist), which suppress the E-cadherin expression. E-cadherin repressors function as EMT inducers on multiple levels, but when cells at the invasive front lose E-cadherin expression, single cell migration occurs [77]. Yet, cell migration in a cohesive group is a hallmark of the tissue remodeling events that underlie embryonic morphogenesis, wound repair and cancer invasion [78]. The mode of sheet migration relies on the cooperative guidance of leader and follower cells throughout the collective group. TGFβ has been shown to stimulate collective migration primarily through extracellular-regulated kinase 1/2 (ERK1/2) activation [79]. On the other hand, using a three-dimensional organotypic culture system, we have described that inhibition of TGFβ signaling increases collection into the underlying extracellular matrix in a fibroblast- and MMP-dependent manner [80]. Additional research has demonstrated that tumor cell knockout of TGFβ signaling, through deletion of the type II receptor, drives fibroblast-stimulated collective migration and metastasis [81].

### 3.3. Wound Healing and Regeneration

Embryogenesis and wound healing enlist similar processes, such as the programmed death of unneeded or damaged cells and cell migration. Wound healing is an elaborate, tightly-regulated process: Briefly, following tissue injury, growth factors and cytokines are released at the wound site. Injured vessels begin to clot due to the deposition of ECM proteins, such as fibronectin and collagen, and form granulation tissue. Over the course of the next several days to weeks, immune cells and fibroblasts infiltrate the granulation tissue, ridding it of debris and rebuilding the matrix [10,12,14,56]. Throughout the process, all cell types require the ability to move, replace and reform tissue. In zebrafish, activin A is required for tissue regeneration following injury, while inhibition of signaling completely blocks regeneration [58,82,83,84]. In a mouse model of wound repair, increased *Inhba*, which encodes the mouse β_A_ subunit, was observed in wound granulation tissue within one day of injury and was sustained for seven days [2,4,6,9,61,85]. As follistatin levels increased concurrently with inhibin β_A_ levels, it has been suggested that the availability of the activin A ligand, not receptor occupation, regulates the wound response [9,26,28,56,85]. Activin A levels become quickly elevated in wounded tissues, likely due to the early inflammatory response [11,13,15,26,63,64,86]. However, as demonstrated in embryogenesis, activin A operates in a concentration-dependent manner. When activin A is overexpressed in the skin, wounds heal more quickly, however being associated with substantial fibrosis [27,61,66,87,88,89].

TGFβ has been highly characterized to promote a reactive stroma [65,89,90]. Similarly to activin A, TGFβ can support all aspects of wound granulation tissue, such as attracting macrophages and fibroblasts to the injury site, initiate wound angiogenesis and stimulate ECM deposition and inflammation [50,67,91]. During wound healing, activin A and TGFβ function in similar roles.

## 4. Contributions to Tumorigenesis

**Figure 3:**
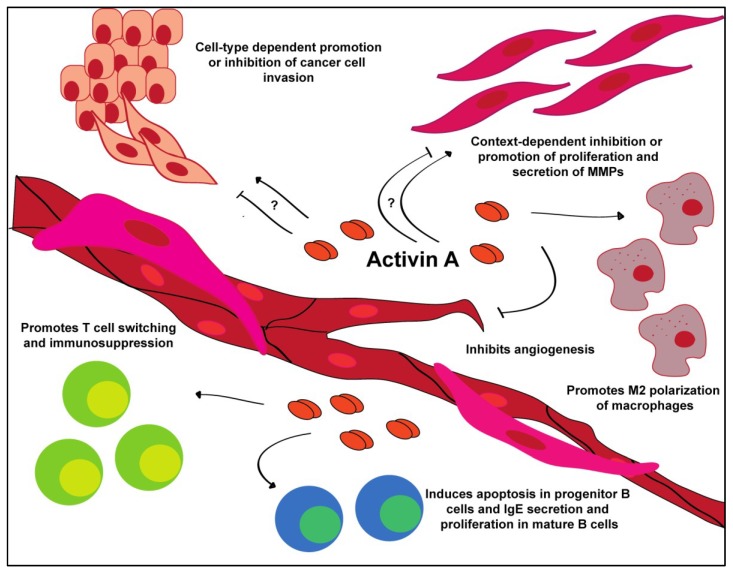
Tumor microenvironmental interactions of activin A. Activin A promotes a variety of behaviors in a cell-type and context-dependent manner.

Harold Dvorak elegantly described cancer as being “the wound that does not heal”. Activin A and TGFβ are excellent examples of this phenomenon, as they both show similar functions in development and wound healing to that observed in cancer initiation and progression. Interestingly, both ligands demonstrate cell type and context-dependent roles within the tumor microenvironment, illustrated below (Figure 3).

### 4.1. Epithelial Tumors

As the role of activin A has been explored in a variety of epithelial tumors, differences in action emerged between cancers. It has been shown that activin A can exert a primarily protective function [51,52,54,68,92]. Experiments utilizing patient-derived prostate cancer cells and non-invasive LNCaP have demonstrated that treatment with activin A results in cell cycle arrest [45,93,94,95]. Interestingly, LNCaP cells showed no response when treated with the exogenous TGFβ ligand [96,97]. In contrast, activin A treatment of the more aggressive prostate cancer cell line PC3 resulted in an increase in proliferation [69,98]. Recent evidence has implicated endoglin, a TGFβ type III receptor and co-receptor, in cancer cell invasion in prostate cancer cell lines via activin A signaling, though endoglin has primarily been shown to propagate the signal by forming a complex with TGFβ and its receptors [99]. Co-expression of endoglin and ActRIIA, when expressed on PC3 or DU-145 cells, has shown suppressed cancer cell invasion, while co-expression of endoglin with ActRIIB, BMP and TGFβ does not exhibit this effect. This functional effect is likely mediated through non-canonical Smad signaling; however, the mechanism of action needs to be further explored [100]. Additionally, TGFβ has an oncogenic effect on PC3 cells, as well as the breast cancer cell line, MDA-MB-231. When acting via the non-canonical MAPK/TRAF6 pathway, TGFβ induces a pro-migratory, invasive phenotype [70,101]. An *in vitro* analysis of 15 breast cancer cell lines detected activin A expression in only four cell lines [59,71,102]. Functionally, when treated with activin A, T47D cells showed the induction of cyclin-dependent kinase inhibitors p21 and p27 and the cell cycle control protein p15^INK4B^, as well as the downregulation of cyclin A, resulting in increased apoptosis and cell cycle arrest. Similarly, MCF7 cells, which have no detectable endogenous activin A, are highly sensitive to the growth inhibitory effects of activin A [1,3,5,7]. In early tumorigenesis, TGFβ has been shown to have a similar effect. In an overlapping pathway to activin A signaling, TGFβ induces cell cycle arrest through the induction of the cyclin-dependent kinase inhibitors p15^INK4B^, p16^INK4A^, p21 and p27 [8,10,12,14]. However, in some cancers, tumor cells lose their ability to respond to the growth inhibitory effects of both activin A and TGFβ. This occurs primarily through mutation or downregulation of the receptor; however, this is not always the case [2,4,6,82,83,84,103].

In contrast to its characteristic growth inhibitory effects, activin A expression has also been associated with inducing an invasive phenotype in certain cancers (Figure 3). In lung adenocarcinoma and oral squamous cell carcinomas, for example, activin A overexpression is correlated with positive lymph node status and poor patient prognosis [9,11,13,15,85,104,105]. In head and neck squamous cell carcinoma, increased activin A has been hypothesized to be an independent prognostic marker of survival [9,26,27,28,85,106]. *In vitro* treatment of the lung cancer cell lines, H460 and SKLU1, with recombinant activin A showed increased proliferation [26,29,30,63,86,107,108,109]. Additionally, treatment with recombinant activin A increased invasion in the ovarian cancer cell lines, SKOV-3 and OCC1, without impacting proliferation [110].

MMP-7, a matrix metalloproteinase capable of degrading many components of the ECM and activating additional MMPs responsible for increased cell invasion, is upregulated in the presence of activin A [31,32,33,34,35,87,88,89,109]. This occurs through c-Jun/Smad activity inducing MMP-7 transcription via the AP-1 promoter region [41,42,43,89,90,111]. Additionally, *in vitro* and clinical evidence suggest that activin A may drive cell invasion by upregulating N-cadherin, a marker of mesenchymal cells and invasiveness [44,45,46,47,50,91,112]. N-cadherin expression is positively correlated with activin A, regardless of E-cadherin expression [48,49,52,92,113,114,115].

In a similar mechanism to activin A, TGFβ has also been shown to promote cancer cell progression. TGFβ is a potent inducer of EMT through its canonical Smad signaling pathway, as demonstrated in various cancer cell types. TGFβ prompts the expression of Snail family zinc finger 2 (Slug), Snail and twist, which act to repress E-cadherin expression [8,45,50,51,58,93,94,103]. EMT can also be induced through TGFβ non-canonical signaling pathways. It has been recently demonstrated that TGFβ can act through TRAF6 to promote receptor cleavage of ALK5/TβRI, which allows for the cleaved intracellular domain to translocate to the nucleus and, in association with p300, drive transcription of various invasion-promoting genes [8,51,52,53,54,116,117]. Increased expression of TGFβ has been noted in various cancers, such as lung, breast and gastric cancers, and has been associated with poor patient prognosis [51,54,55,96,118]. Additionally, TGFβ can stimulate and alter MMP expression from epithelial cells. Several groups have shown that TGFβ can negatively regulate MMP-1 and MMP-7 through canonical Smad signaling, while stimulating the production of MMP-2 and MMP-9 through non-canonical p38 and NFkB signaling pathways [1,37,49,98,119]. MMP production and activation are necessary for degrading ECM components, allowing for cell migration and further invasion into the stroma.

When overexpressed in the tumor, activin A confers differential effects. Some cancers, such as lung and head and neck squamous cell carcinoma, develop insensitivity to the growth inhibitory effects of activin A, one of the hallmarks of cancer [56,57,101,120]. During cancer progression, cells in the tumor microenvironment, such as T-helper 1 (T_h_1) cells and fibroblasts, increase their production of activin A in an attempt to inhibit the growth of the tumor; however, the cancer cells adapt and evade these signals. Tumor cells that show resistance to activin A may do so by downregulating ALK4, which is responsible for signal transduction, or by upregulating follistatin or inhibin A production; however, these areas need to be further explored [58,59,102,121].

### 4.2. Immune Cells

Activin A plays a key role in the maturation and activation of the innate and adaptive immune systems (Figure 3). In the normal immune response, activin A is on the forefront of fighting infection [1,2,4,5,6,60,61,122]. In the humoral immune response, activin A is secreted by and plays a significant role in the function of adaptive immune cells. In the T-cell population, activin A is secreted specifically by activated CD4+CD25- (CD25: interleukin 2 receptor α) T-helper 2 (T_h_2) cells [8,56,62,123,124]. However, activin A also contributes to the switching of CD4+CD25-Foxp3- (Foxp3: forkhead box P3) cells to CD4+CD25+Foxp3+ T-regulatory (T_reg_) cells, which correlates with immunosuppression in patients with B-cell acute lymphoblastic leukemia [11,13,15,26,63,64,103,125]. T_reg_ cells downregulate the actions of T_h_1, T_h_2 and T-helper 17 (T_h_17) cells, limiting their ability to recognize and potentially destroy cancer cells. Similarly, TGFβ induces a similar Foxp3+ T_reg_ cell phenotype [9,27,61,66,105,126,127]. Together, T-cells induced by either activin A or TGFβ promote a pro-tumor microenvironment.

Activin A induces stage-dependent effects on B-cells [9,26,28,65,106,127]. This has been demonstrated in a variety of studies, where exogenous treatment of B-cells derived from marrow stem cells, cultured cell lines and mature B-cells can induce apoptosis in the former and proliferation and antibody secretion in the latter [29,65,67,107,108,109]. Additionally, activin A is secreted by activated B-cells, which, in turn, stimulates the production of IgE antibodies [31,51,54,68,109,128,129]. TGFβ performs a similar function as activin A and inhibits the proliferation of progenitor B cells; however, it also has the ability to induce growth arrest in B maturation [41,45,95,111]. Conversely, TGFβ can drive B-cell differentiation and stimulate the production of IgE and, in some cells, IgG [44,46,47,51,53,54,112,130,131].

The effect of activin A in the immune system has been most heavily studied in the macrophages, though activin A has also been shown to affect mast cells, natural killer cells and dendritic cells [48,97,113,114,115]. As discussed above, activin A is secreted by T_h_2-helper cells, which also secrete high levels of interleukin-4 (IL-4) and interleukin-13 (IL-13). IL-4 and IL-13 promote the alternative activation pathway of macrophages, M2, that is commonly associated with wound healing and cancer [8,50,58,69,103]. Interestingly, *in vitro* activin A stimulation promoted the M2 macrophage phenotype, suggesting its functional similarities to IL-4 and IL-13 in tumor promotion [8,52,70,117]. TGFβ also polarizes macrophages to the alternative M2 phenotype, promoting an immunosuppressive microenvironment [55,59,71,118]. These M2 macrophages secrete cytokines and MMPs that promote a favorable tumor microenvironment [132].

Condeelis and Pollard, in their review of the multifaceted nature of macrophages, succinctly stated that “tumors recruit macrophages and create a microenvironment that causes macrophages to suppress immune functions and, instead, adapt trophic roles found during development and repair” [1,3,7,37,119]. This involves creating a favorable environment for tumor cell invasion and recruitment of fibroblasts and endothelial cells to the microenvironment. For example, secretion of interleukin-2 and interferon-α from T-cells drive the release of basic fibroblast growth factor, leading to the subsequent induction of endothelial cell and fibroblast migration [36,120]. Activin A and TGFβ play important roles in this process, reasserting developmental function in the incorrect context, and promote pathogenesis.

### 4.3. Fibroblasts

Fibroblasts in the tumor microenvironment contribute to the creation of a reactive stroma or the transformed state of the stroma in response to disrupted homeostasis, wounding or cancer initiation [72,121]. In normal physiology, fibroblasts typically express activin A at negligible levels [73,122]. Activin A stimulates proliferation of mouse 3T3 fibroblasts, airway smooth muscle cells and lung fibroblasts [74,123,124]. Interestingly, we have shown that overexpression of activin A by normal esophageal fibroblasts in a pre-neoplastic microenvironment inhibits proliferation in both an autocrine and paracrine manner; however, it switches to a tumor promoter in the presence of malignant cells [133]. A similar phenotype has been noted in the context of TGFβ signaling [75,126].

Tumor-associated myofibroblasts (TAMs) of oral squamous cell carcinoma, which express markers of activation (e.g., α-smooth muscle actin (αSMA), platelet-derived growth factor-α (PDGFα), fibroblast activating protein (FAP)), secrete increased levels of activin A [76,127,128]. This was associated with increased secretion of collagen I, MMP-1, MMP-2, MMP-9 and MMP-13, as well as increased proliferation and *in vivo* tumor volume [77,128]. In comparison, the development of a reactive stroma has been correlated with increased secretion of TGFβ by pre-neoplastic cells [65,78]. However, it has been consistently shown that fibroblasts that lose responsiveness to TGFβ promote collective cell invasion [79,129,130]. These results suggest that, in contrast to activin A, TGFβ signaling in the tumor stroma is necessary to maintain an intact microenvironment and prevent tumor cell invasion, though it has been suggested that TGFβ may act through the reactive stroma, not the epithelial cell compartment, to promote tumor angiogenesis [2,4,6,80,134]. This is a significant point, where the functional consequences of TGFβ and activin A diverge.

TAMs secrete proinflammatory cytokines and proteases to drive EMT and proliferation and migration of epithelial and endothelial cells [11,13,15,81,135,136]. These results suggest that TAMs can prime the tumor microenvironment for epithelial cancer cell invasion by rearranging the ECM. Additionally, TAMs have been associated with the recruitment of immunosuppressive cells to the tumor front [10,12,14,27,56,98]. The localization of myofibroblasts to the tumor has been indicative of poor patient prognosis [30,58,82,83,84,137,138]. The recruitment and activation of fibroblasts at the tumor front results in an aggressive and invasive phenotype, as tumor cells use fibroblasts to alter the ECM.

### 4.4. Endothelial Cells

In contrast to its tumor promoting function in immune cells, activin A has been consistently shown to operate as a potent anti-angiogenic factor (Figure 3). Treatment of endothelial cells demonstrated decreased tube formation and inhibition of proliferation via induction of p21 and decreased expression of cyclin D1 and Rb [2,4,6,9,32,33,34,35,61,85,139,140]. This effect can be overcome by silencing of p21. Additionally, fibroblast-derived overexpression of activin A downregulates vascular endothelial growth factor (VEGF) expression, one of the key components of tumor angiogenesis [133]. Previous research has shown that most endothelial cells express ActRII/IIB and are therefore able to respond to activin A ligand binding [9,26,28,42,43,56,85,140]. Activin A treatment of endothelial cells results in a dose-dependent inhibition of proliferation. Experiments utilizing chick chorioallantoic membrane demonstrated a complete loss of capillary development and fibrosis when treated with activin A [11,13,15,26,45,63,64,86,141]. In our experiments, conditioned media from normal esophageal fibroblasts that overexpress activin A completely inhibits the tube formation of endothelial cells [133]. *In vivo*, breast cancer cells expressing activin A develop larger, though less vascularized, tumors compared to cells expressing follistatin, which form smaller, better vascularized tumors [27,49,61,66,87,88,89,142].

Due to its potent anti-angiogenic nature, cancer cells have developed mechanisms to counteract activin A activity. Activin A expression in neuroblastoma results in elevated cyclin-dependent kinase inhibitors and decreased vascular endothelial growth factor receptor 2 (VEGFR2) [45,51,65,89,90,143]. Therefore, the oncogene N-myc, which is consistently overexpressed in neuroblastoma, stifles this effect by directly inhibiting transcription of the inhibin β_A_ subunit and, subsequently, activin A homodimer formation [50,51,53,54,67,91,141]. Additionally, activin A may be negatively regulated by interleukin-32 (IL-32), which promotes proliferation and endothelial cell function during tumor promotion. This effect is overcome with the silencing of IL-32, which results in the upregulation of activin A [51,52,54,68,92,144].

TGFβ has been consistently shown to both induce and inhibit angiogenesis. In contrast to activin A’s function, TGFβ directly stimulates the pro-angiogenic protein, vascular endothelial growth factor A (VEGF-A), via Smad3, while the angiogenesis inhibitor, thrombospondin-1 (THBS1), is induced via phosphorylated Smad2 [45,49,93,94,95,145,146]. These separate signaling pathways suggest differential effects of TGFβ signaling in cancer development and metastasis. Interestingly, TGFβ-induced angiogenesis can be blocked through treatment with TGFβ inhibitors, latent activating protein (LAP) and BMP, and activin membrane-bound inhibitor (BAMBI) [57,96,97,134,147]. It may be suggested that TGFβ promotes endothelial cell migration and tube formation via a VEGF-dependent pathway, as it is well established that VEGF is a potent driver of tip cell migration [59,69,98,148]. However, recent research has shown that cells’ TGFβ has the ability to bind to ALK1 and ALK5 on the endothelium, which may then dimerize with endoglin to activate Smad1/5/8, the typical BMP signaling pathway, and inhibit endothelial cell proliferation; however, these results remain controversial [149,150].

## 5. Conclusions and Future Directions

As discussed above, activin A plays pleiotropic roles in basic physiology and pathogenesis. Normal activin A function induces embryonic cell fate, wound healing and inhibits proliferation. During tumorigenesis, activin A acts as a suppressor of cancer angiogenesis, as a promoter of tumor-associated macrophages and T-cells and exerts mixed effects on epithelial tumor cells, further exemplifying the context- and cell type-dependent effects of activin A signaling. Based on the evidence presented, the overall functional consequences of activin A signaling alone are not sufficient to either suppress or drive cancer progression and, therefore, must work in collaboration with other pathways to dictate a particular phenotype. This may include working in cooperation with other TGFβ ligands, such as BMP4, or synergistically with other pathways, such as MAPK/ERK [37,59,62,71,102,151]. Further investigation into the mechanism of activin A signaling and the intertwining of different pathways to promote cancer progression is needed to unravel the complex signaling processes.

## Abbreviations

TGFβ: transforming growth factor β;
BMP: bone morphogenic protein;
ALK1: activin receptor-like 1;
ALK4: activin receptor-like kinase 4, ActRIB;
ALK5: activin receptor like kinase 5, TβRI;
ActRII: activin receptor type II;
ActRII: activin receptor type II/IIA;
MMP: matrix metalloproteinase;
*INHBA*: the inhibin βA gene, which encodes the activin βA subunit.
